# Host-specific microbiomes of blow flies: ecological drivers and implications for pathogen carriage

**DOI:** 10.3389/fimmu.2025.1673934

**Published:** 2025-11-27

**Authors:** Aram Mikaelyan, Joseph Receveur, Kadie Bernstein, Nicholas J. Babcock, Jennifer L. Pechal, Michael V. Welsh, Kelly A. Waters, Katherine H. Yoskowitz, M. Eric Benbow

**Affiliations:** 1 Department of Entomology and Plant Pathology, North Carolina State University, Raleigh, NC, United States; 2 Institute for Genome Sciences, University of Maryland, Baltimore, Baltimore, MD, United States; 3 Department of Entomology, Michigan State University, Building, East Lansing, MI, United States; 4 Ecology, Evolution, and Behavior Program, Michigan State University, East Lansing, MI, United States; 5 Department of Osteopathic Medical Specialties, Michigan State University, East Lansing, MI, United States

**Keywords:** blow fly, microbiome, ignatzschineria, vagococcus, pathogen carriage, myiasis, pathogen transmission

## Abstract

Blow flies (*Lucilia sericata* and *Phormia regina*) are necrophagous insects that interact with dense microbial reservoirs and are opportunistic vectors of human and animal pathogens. Despite constant exposure to diverse environmental microbes, it is unclear whether their bacterial communities are primarily acquired stochastically or shaped by host factors that could influence pathogen carriage. We conducted a systematic comparison of wild *L. sericata* and *P. regina* collected from seven cities across an urban-rural gradient to determine whether microbiome composition is structured by host species identity or environmental variables. Using 16S rRNA gene sequencing of individual flies, we profiled bacterial communities and applied alpha- and beta-diversity analyses, PERMANOVA, and Random Forest classification to quantify species-level microbiome differentiation. Species identity was the strongest predictor of microbiome composition (PERMANOVA, *p* = 0.001), while location, land cover type, sampling month, and sex had no significant effects. Random Forest modeling identified multiple bacterial taxa that consistently distinguished the two species, including *Ignatzschineria* and *Dysgonomonas*, which were enriched in *P. regina*, and *Vagococcus* and *Escherichia-Shigella*, which were enriched in *L. sericata*. These taxa are of clinical relevance, with *Ignatzschineria* in particular increasingly reported from human myiasis and soft-tissue infections, sometimes exhibiting antimicrobial resistance. Our findings demonstrate that wild blow flies maintain species-specific microbiomes despite shared environments, suggesting that host identity strongly filters microbial communities. The presence of opportunistic pathogens within these structured microbiomes underscores the need to understand how blow fly–microbe associations contribute to pathogen persistence and dissemination. By revealing predictable, species-dependent microbiome patterns, this study highlights potential targets for microbiome-based strategies aimed at mitigating blow fly–associated disease risks.

## 1 Introduction

Blow flies (Diptera: Calliphoridae) are crucial players in decomposition, and interact with dense microbial reservoirs throughout their life cycle (1). As founding members of necrobiomes, they are among the first insects to arrive at decomposing animal remains, exploiting nutrient-rich environments for feeding and reproduction that are densely colonized by microbes (2). During adult foraging, blow flies land on decomposing carrion (3, 4), fecal matter (5), open wounds (6, 7), and flowers (8, 9), acquiring and depositing bacteria through direct contact and regurgitation-mediated feeding (10). Because blow flies can act as opportunistic vectors of human and animal pathogens (1), understanding whether their microbiomes are shaped by host identity or by stochastic environmental acquisition is critical for assessing disease transmission risk.

Despite their exposure to fluctuating microbial landscapes, blow flies appear to associate with certain bacterial taxa more consistently than would be expected under purely stochastic environmental acquisition (11, 12). This suggests that underlying host factors – whether physiological, immune-mediated, or behavioral – may play a role in shaping microbiome composition. Distinguishing between these mechanisms is critical for addressing broader ecological and applied questions: Do certain blow fly species harbor microbiomes with enhanced decomposition-promoting capabilities? Could species-specific microbiomes alter how blow flies acquire and spread pathogens? And could these differences be factored into forensic use?

While existing studies provide some evidence that blow fly microbiomes are dominated by bacterial members primarily from the phyla Proteobacteria and Firmicutes, it remains unclear whether this specificity holds at finer and more functionally relevant taxonomic levels. Despite *Lucilia sericata’s* (Meigen 1826) being one of the most extensively studied blow fly microbiomes, previous work has not resolved whether it maintains a species-specific microbiome. Instead, methodological differences among studies have resulted in highly variable microbiome compositions, raising fundamental questions about how much of a blow fly’s microbiome is truly host-determined and how much is influenced in which the fly develops or forages. For instance, three independent studies that reared *L. sericata* under controlled conditions yielded dramatically different microbiome profiles. Iancu et al. (13) found that flies raised on swine liver were overwhelmingly dominated by *Lactobacillus*, while Singh et al. (14) and Wohlfahrt et al. (11), who reared flies on beef liver, reported *Providencia* as the dominant genus instead.

More discrepancies emerge when comparing microbiomes of wild-caught and lab-reared individuals of the same species. For instance, lab-reared *Phormia regina* (Meigen, 1826; 11) was dominated by *Providencia* and *Vagococcus*, whereas wild-caught *P. regina* (15) contained *Dysgonomonas* and *Ignatzschineria*, both of which have been recovered from clinical infections, including myiasis-associated cases. The New World Screwworm *Cochliomyia hominivorax* (16), an obligate parasite that feeds on the living tissue of mammals, showed a similar contrast, with lab-reared flies dominated by *Providencia* and *Vagococcus*, while wild-caught individuals harbored *Streptococcus* and *Fusobacterium*. One likely explanation is that flies reared on fresh meat in the lab lack access to the complex microbial reservoirs encountered in their natural habitats and variable carrion resources, limiting the generalizability of laboratory-based microbiome studies.

These discrepancies highlight a fundamental gap: without systematic sampling of wild blow flies across habitats and time, we cannot determine whether their microbiomes are truly host-specific. The only way to begin resolving this is through longitudinal sampling of wild populations, paired with rigorous environmental and host-based comparisons.

This study aims to determine whether blow fly microbiome composition is primarily structured by species identity or by environmental factors, a key step toward assessing their potential role in pathogen transmission. It builds upon the framework established by Babcock et al. (17), extending their ecological survey of blow fly species across urban-rural gradients to examine the bacterial communities within individual flies to identify the factors – particularly host species identity – that structure their microbiomes. Specifically, we ask – using *L. sericata* and *P. regina* – the following: (1) Do individuals of these two species harbor distinct microbial communities, regardless of environmental context? and (2) How resilient are their microbiomes to variation across urban-rural gradients and seasonal shifts?

We conducted a systematic comparison of *L. sericata* and *P. regina* across diverse environmental conditions. By sampling wild populations from seven cities across an urban-rural gradient, we assessed whether microbiome composition is shaped primarily by host identity or environmental exposure. We employed high-throughput sequencing of the 16S rRNA gene to profile microbiomes at high taxonomic resolution and quantified species-level microbiome structuring while controlling for multiple factors. In addition to standard diversity analyses, we used Random Forest modeling to identify the microbial taxa that best predict variation across multiple metadata categories associated with our sampling, allowing us to assess whether clinically relevant taxa are consistently associated with particular blow fly species.

## 2 Methods and materials

### 2.1 Study area and blow fly collection

Blow flies were collected from Mid-Michigan (USA) across seven cities: Mason, Lansing, Perry, DeWitt, Grand Ledge, Charlotte, and Williamston. These sites were selected to broadly represent urban-rural land cover as defined in the National Land Cover Database (NLCD) and were chosen based on accessibility within urban settings and locations approximately 10 km outside of the urban center, following a prior study of blow fly community composition in the region (17). The City of Lansing was the only site without rural land use comparisons. In total, 33 *L. sericata* and 32 P*. regina* individuals were analyzed for microbiome composition across these sites.

Blow flies were captured using inverted cone traps, which were constructed from plywood and polyester mosquito netting (17). Each trap measured 76.2 cm in length and 25.4 cm in width and was baited with decomposing beef liver. The traps allowed flies to enter but prevented escape. Sampling occurred during the summer months of June, July, and August 2017, with collections conducted once per month at each site, with additional details of the site locations, methods and specimen identification found in Babcock et al. (17). All individuals were wild-caught adults collected directly from these traps. Species identity was confirmed morphologically using diagnostic characters of 18, and sex was determined morphologically based on eye placement on the head (e.g., holoptic vs dichoptic; 19, 20). Collected flies were transported to the laboratory and stored at -20°C until further processing.

### 2.2 DNA extraction and amplicon sequencing

Individual flies were rinsed in 10% bleach, followed by two rinses in sterile deionized water for surface sterilization and nucleic acid decontamination, as previously described (21), to reduce external microbial carryover and optimize for the internal fly microbiomes. Genomic DNA was extracted using the Qiagen DNeasy Blood and Tissue Kit (Valencia, CA, USA) following a modified manufacturer protocol, in which lysozyme (15 mg/ml) was added during the lysis step to enhance bacterial cell wall disruption. DNA concentrations were quantified using the Qubit 1x dsDNA HS Assay Kit and a Qubit 2.0 fluorometer (Thermo Fisher Scientific, Grand Island, NY, USA). Samples were stored at -20 °C until sequencing.

Microbiome characterization was performed using 16S rRNA gene amplicon sequencing, targeting the V4 region with the 515F/806R primer set (22). Library preparation and sequencing were conducted at the Michigan State University Genomics Core Facility (East Lansing, MI, USA) on an Illumina MiSeq platform (2 × 250 bp paired-end reads with unique dual barcoding indices).

### 2.3 Bioinformatics and data processing

Raw sequencing reads were processed using BBMerge to merge paired-end reads. Quality-trimming and sequence processing were done using Mothur (v.1.47.0), following a customized pipeline adapted from the MiSeq SOP (23). Sequences were only retained if they had no ambiguous base calls, no homopolymer runs greater than 10 bases, were at least 200 bases in length, and passed an average quality threshold of 25, with a sliding window of 50 bases. Identical sequences were collapsed using unique.seqs, and abundance information was tracked using count.seqs. Unique sequences were then aligned to the SILVA reference alignment (v138.1). Chimeric sequences were detected and removed using the VSEARCH algorithm (v2.21.1). Sequences were analyzed at the unique-sequence level (i.e. non-redundant sequences generated by unique.seqs that each represent a distinct sequence variant in the dataset), without clustering into OTUs. Samples with fewer than 1,000 total reads classified to the genus level were excluded from downstream analyses.

Taxonomic classification was conducted using the SILVA database (v138.1) at the genus level. Sequences matching non-target lineages – such as mitochondria, chloroplasts, *Blattabacterium*, *Wolbachia*, or those unclassified at the domain level – were removed. The resulting count tables were converted to relative abundance for downstream statistical analyses.

### 2.4 Statistical analyses

#### 2.4.1 Normalization of sequence data

Samples with fewer than 1,000 total reads were excluded from all downstream analyses to minimize biases associated with low sequencing depth. For the remaining samples, counts were normalized by total-sum scaling to a constant library size of 1,000 reads per sample and subsequently converted to relative abundances. This standardized approach was applied consistently across analyses, including β-diversity (PERMANOVA and PCoA), Random Forest classification, and Tweedie generalized linear modeling, ensuring comparability among samples without the need for rarefaction.

#### 2.4.2 Alpha diversity analysis

Alpha diversity was measured using the Shannon diversity index, which accounts for both species richness and evenness within each sample. Shannon index values were calculated from the relative abundance matrix using the diversity() function in the vegan R package (24). To determine whether alpha diversity varied significantly across sample groups, the Shannon diversity index was compared among Species (*L. sericata* vs. *P. regina*), City (Mason, Lansing, Perry, DeWitt, Grand Ledge, Charlotte, Williamston), Location (urban, rural), Month (June, July, August), and Sex (Male, Female). To test for any influence of sample processing (which was performed by nine individuals), we included an additional factor (“Processor”). The variable Date was omitted because it was collinear with Month and thus redundant in the statistical models.

Before statistical testing, Shapiro-Wilk normality tests were conducted to determine whether Shannon index values were normally distributed within each metadata category. Since the data were not normally distributed, non-parametric tests were used for all comparisons. For binary comparisons (e.g., species, sex), the Wilcoxon rank-sum test (Mann-Whitney U test) was applied. For multi-group comparisons (e.g., city, location, month), the Kruskal-Wallis test was used. Statistical significance was determined at *p* < 0.05, and *p-*values were adjusted for multiple comparisons where applicable.

#### 2.4.3 Beta diversity analysis

Beta diversity was assessed at the bacterial genus level using two ecological distance metrics, the Morisita-Horn index and the Jaccard index, which were computed from the relative abundance matrix. The Morisita-Horn index was used to calculate the degree of similarity among communities weighted by species abundance, while the Jaccard index, a non-weighted presence/absence-based metric, was used to measure community similarity based on the number of shared taxa, without considering their relative abundances. The Morisita-Horn index was selected over Bray-Curtis for its robustness to differences in sampling depth and stronger emphasis on dominant taxa. The indices were computed using the vegdist() function in the vegan R package with the “horn” and “jaccard” methods (24).

Principal coordinate analysis (PCoA) was conducted to visualize microbiome composition differences among samples calculated using both metrics. The plot generated using the cmdscale() function in R (v4.1.2) via the vegan package was color-coded by species. To incorporate alpha diversity (Shannon index) into the PCoA plots, sample points were scaled to the Shannon index, with larger points indicating higher within-sample diversity.

Permutational multivariate analysis of variance (PERMANOVA) was conducted using the adonis2() function in vegan (24) to determine the effects of multiple metadata variables on microbiome composition. The model included the factors City, Location, Month, Species, and Sex, and was run separately for both Morisita-Horn and Jaccard distance matrices. The variable date was omitted due to its collinearity with Month. Nested PERMANOVA analyses were conducted, stratifying by species, to further investigate within-species variation.

#### 2.4.4 Random forest analysis of important microbial taxa

Random Forest classification was performed in R (25) using the randomForest package (500 trees per model) to identify which bacterial taxa best distinguished samples according to seven metadata variables (Species, City, Location, Type, Month, Processor, and Sex). For each variable-specific model, we recorded its out-of-bag (OOB) accuracy – a built-in estimate of how well the classifier can predict unseen samples – and extracted each taxon’s Mean Decrease in Gini (MDG), which quantifies how much that taxon reduces classification error at each split. Using MDG alone can disproportionately emphasize taxa in models with low predictive power, while OOB accuracy alone overlooks how individual taxa contribute to model discrimination. Therefore, we weighted each taxon’s importance by multiplying its MDG score by the model’s OOB accuracy to mitigate biases arising from poorly performing models to calculate the taxon contribution score, which prioritizes taxa that reliably distinguish sample categories. Finally, we summed taxon contribution scores for each bacterial taxon across all metadata classifiers.


$$TCS_{t,m}=\;MDG_{t,m}\;\times \;OOB_{m}\;$$

$$cTCS_{t}=\sum_{m\;\in \;M}\left(TCS_{t,m}\right)$$

Here, *MDG_t,_* is the Mean Decrease in Gini for taxon *t* in classifier *m*, *OOB_m_* is the out−of−bag accuracy of classifier *m*, and *M* is the set of all metadata classifiers. *TCS_t_* represents the weighted importance of taxon *t* in classifier *m*, and *cTCS_t_* is the cumulative score summed across all classifiers.

The resulting cumulative taxon contribution score allowed us to identify taxa that consistently distinguished sample categories across multiple variables, and were used to rank taxa and select the top 30 for visualization with the pheatmap package (26) in *R*.

To assess differences in the relative abundance of the 30 bacterial taxa between *L. sericata* and *P. regina*, we employed a Tweedie Generalized Linear Model (GLM). The Tweedie model is well-suited for microbial community data due to its ability to handle zero-inflated, non-normally distributed, and overdispersed count data without the need for artificial transformations or zero imputation. The model was implemented in R using the glm() function from the statmod (27) and tweedie (28) packages. A Wald test was used to determine the significance of the species effect in each model. False Discovery Rate (FDR) correction (Benjamini-Hochberg method) was applied to account for multiple comparisons across taxa. Taxa with adjusted *p-*values < 0.05 were considered significantly different between species. If a taxon was significantly different, it was classified as enriched in *L. sericata* or *P. regina* based on the mean relative abundance.

## 3 Results

### 3.1 Alpha diversity

Quality trimming of the assembled contigs revealed that the samples had a median of 35,670 reads (*IQR*: 8,629–47,859); samples with fewer than 1,000 reads assigned at the genus level were excluded. Of the 65 initial samples that were sequenced, 60 passed quality filtering and were included in downstream analyses (**Supplementary Table S1**). Shannon diversity index values were compared across metadata variables, including (fly) Species, City, Location, Month, (landcover) Type and Sex, using Wilcoxon rank-sum or Kruskal-Wallis tests as appropriate. Species identity was the only factor that significantly influenced alpha diversity (Wilcoxon *p* = 0.0039; **Supplementary Table S2**), with *L. sericata* exhibiting a higher median Shannon index (1.19; IQR 0.73–2.00; range 0.31–3.92) than *P. regina* (0.79; IQR 0.42–1.14; range 0.11–2.87; **Supplementary Figure S1**). In contrast, none of the other metadata variables (city, location, month, land-cover type, or sex) significantly affected Shannon diversity (all *p* > 0.26). These results indicate that host species was the primary driver of genus-level bacterial diversity in *Lucilia sericata* and *Phormia regina*, whereas external environmental and temporal factors had minimal impact on bacterial richness and evenness.

### 3.2 Beta diversity and principal coordinate analysis

**Figure 1:**
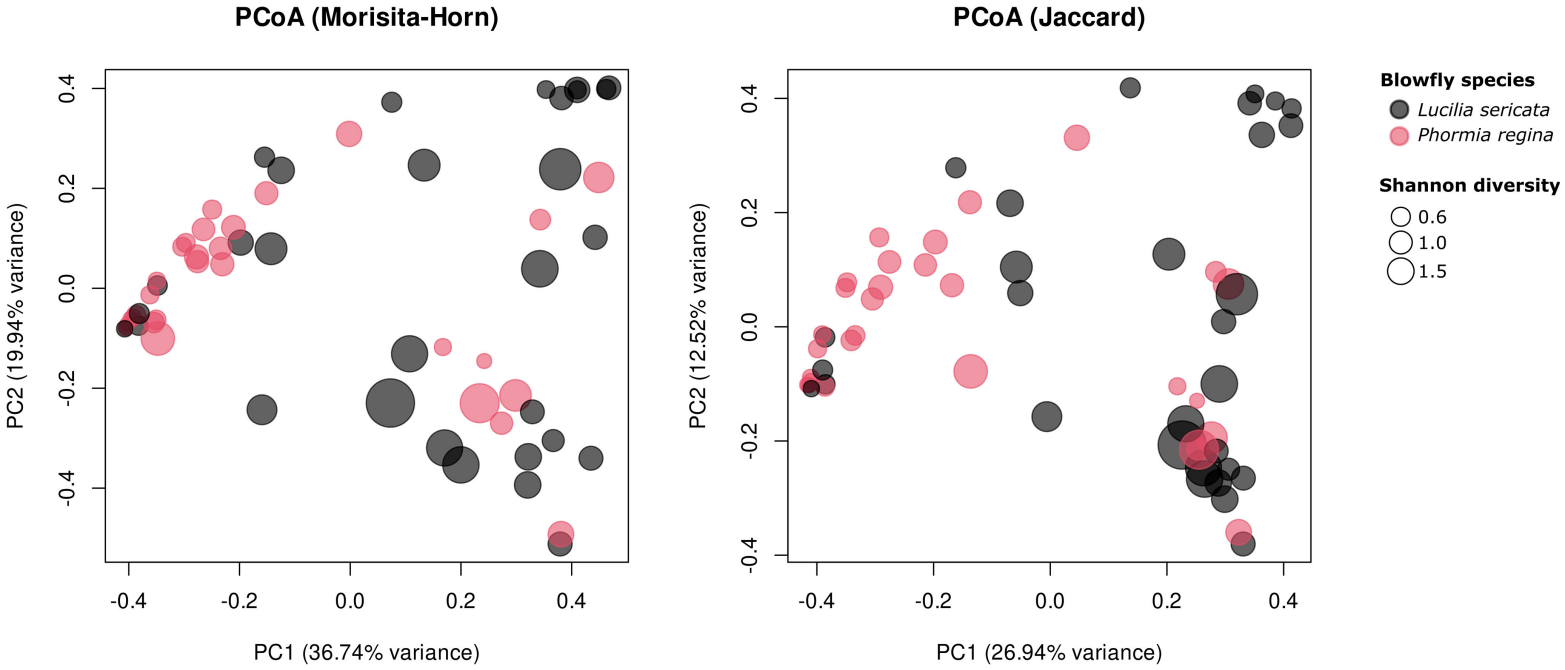
Principal coordinate analysis of blow fly microbiome composition using (left panel) Morisita-Horn and (right panel) Jaccard distance metrics. Each point represents an individual sample, colored by species and scaled by Shannon diversity (larger points indicate higher diversity). Axes show the first two principal coordinates and the percentage of total variation they explain.

Principal coordinate analysis (PCoA; **Figure 1**) revealed distinct clustering of *L. sericata* and *P. regina*, confirming that species identity is a strong driver of microbiome variation (**Figure 1**). To examine the factors influencing community composition, we conducted a beta diversity analysis based on the Morisita-Horn and Jaccard distance metrics. Our PERMANOVA results (**Supplementary Table S3**) indicated that species identity was the single most important driver of microbial community differences, while environmental and demographic factors played a minimal role. Among all tested variables, species identity was the only significant predictor of beta diversity (Morisita-Horn: R² = 0.109, F_1,58_ = 7.06, *p* = 0.001; Jaccard: R² = 0.088, F_1,58_ = 5.61, *p* = 0.001), suggesting that differences in microbial composition are largely dictated by host species rather than external conditions. In contrast, location and landcover type (urban vs. rural) did not significantly influence beta diversity (*p*-values exceeding 0.18 across both metrics). Likewise, sampling month had no discernible impact on community composition (*p* > 0.56) or structure (*p* > 0.34). Similarly, Sex and Processor did not significantly affect fly microbiome community composition (*p* > 0.10). Supplementary PCoA plots annotated by City, Species, Type, and Month (**Supplementary Figure S2**) showed no visible clustering along these environmental gradients, consistent with the PERMANOVA results.

### 3.3 Taxonomic importance across metadata variables

Out‐of‐bag accuracies for the six classifiers ranged from 0.20 (Location) to 0.82 (Type), with Species at 0.72, City and Sex both at 0.53, and Month and Processor at 0.40. Higher OOB accuracies reflect how well each metadata variable can be predicted from the microbiome – higher accuracy indicates the microbial communities differ more distinctly between that variable’s groups. We quantified each taxon’s Mean Decrease in Gini (its contribution to reducing node impurity) for the Species classifier and multiplied these values by the model’s out‐of‐bag (OOB) accuracy to generate a weighted taxon contribution score, prioritizing taxa that both strongly distinguish the two fly species and occur in more accurate models.

**Figure 2:**
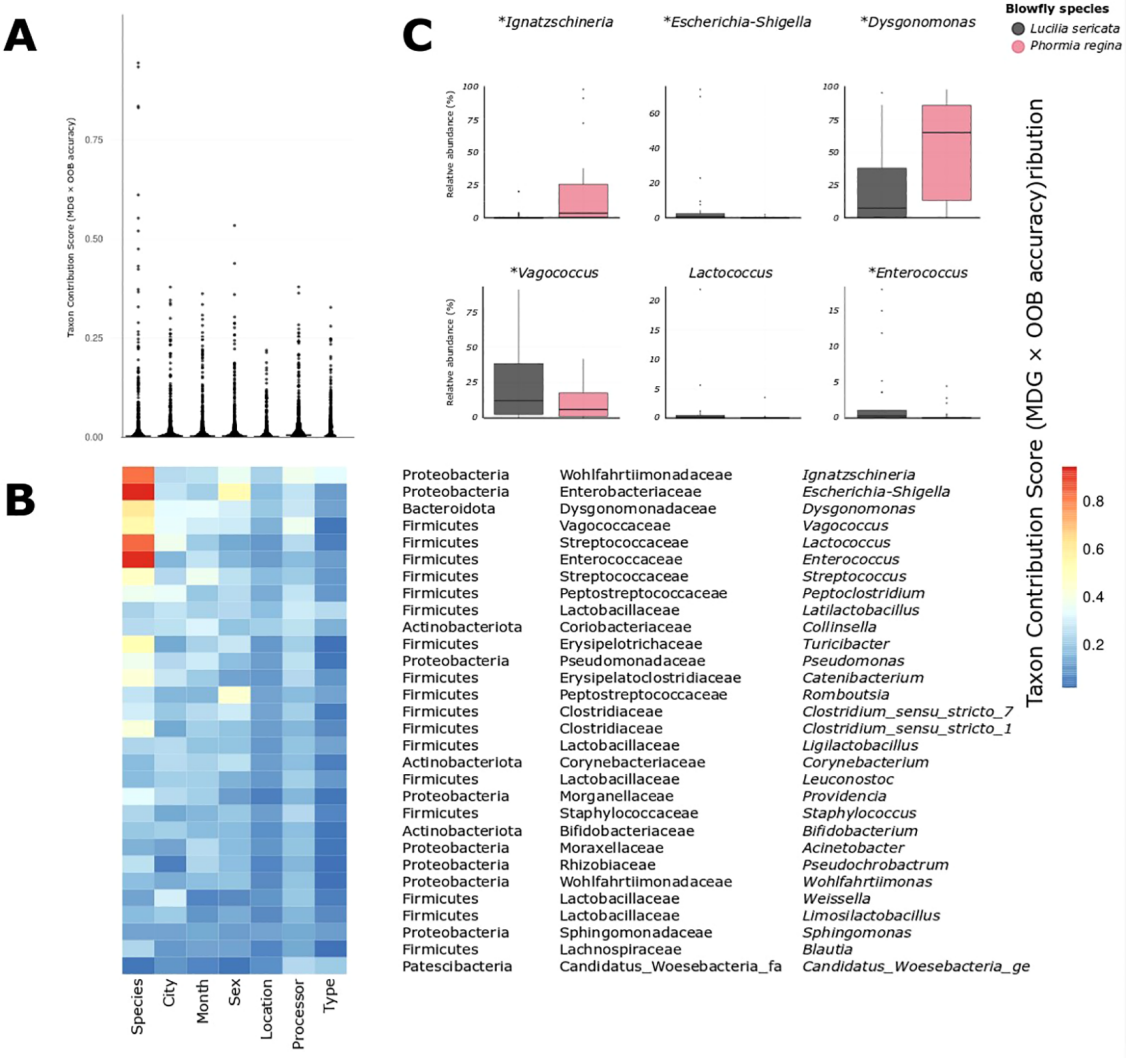
**(A)** Distribution of taxon contribution scores (Mean Decrease Gini × OOB accuracy, summed across all classifiers) for all bacterial taxa, grouped by metadata variable. Each point represents a taxon's importance for distinguishing a given metadata variable given in **(B)**. **(B)** Heatmap showing the same taxon contribution scores for the top 30 most important taxa given in B. Every taxon shows its strongest signal under the "Species" classifier, supporting the unique explanatory power of species identity. **(C)** Relative abundance distributions for six of the most predictive genera (including *Ignatzschineria*, *Dysgonomonas*, *Vagococcus*, and *Escherichia-Shigella*), all of which differ between species. Asterisks denote statistically significant differences based on Tweedie GLM (*p* < 0.05).

To evaluate the extent and distribution of a microbiome signal across metadata variables, we first plotted the taxon contribution scores (Mean Decrease in Gini × out-of-bag accuracy) for every genus-level taxon across all classifiers (**Figure 2A**). Across most metadata classifiers, the distribution of the scores was sparse and clustered near zero, indicating that only a small subset of bacterial genus-level taxa meaningfully contributed to the classification of variables such as City, Month, or Type. In contrast, the distribution for the Species classifier was broader and more continuous, with a notably higher number of taxa exhibiting elevated scores, implying that microbial differentiation between *L. sericata* and *P. regina* is not only more pronounced but also represented by several bacterial taxa (**Figure 2A**).

This pattern is visually reinforced in the heatmap of the top 30 taxa (**Figure 2B**): although these taxa were selected based on cumulative importance across all classifiers, nearly all show their strongest contribution under the Species classifier. This list spanned multiple phyla, including Proteobacteria, Firmicutes, Bacteroidota, Actinobacteriota and Patescibacteria, with the highest cumulative scores observed for *Ignatzschineria*, *Escherichia-Shigella* and *Dysgonomonas*. Consistently, every one of the top 30 taxa had its strongest weight under the Species classifier, underscoring that host identity is by far the main axis of microbiome differentiation.

### 3.4 Differentially abundant bacterial taxa between species

The top 30 genus-level groups retained for detailed comparison accounted for a median 96.7% of reads in *Lucilia sericata* samples (IQR: 85.4 – 99.0%) and 99.4% in *Phormia regina* (IQR: 98.8 – 99.8%), confirming that they captured nearly the entire community in both hosts. Although the Random Forest model for Type (urban vs. rural) achieved the highest OOB accuracy (0.82), this did not correspond to significant differences in relative abundance between urban and rural collections, suggesting that the underlying microbiome composition of these taxa is not driven by these habitat.

**Figure 3:**
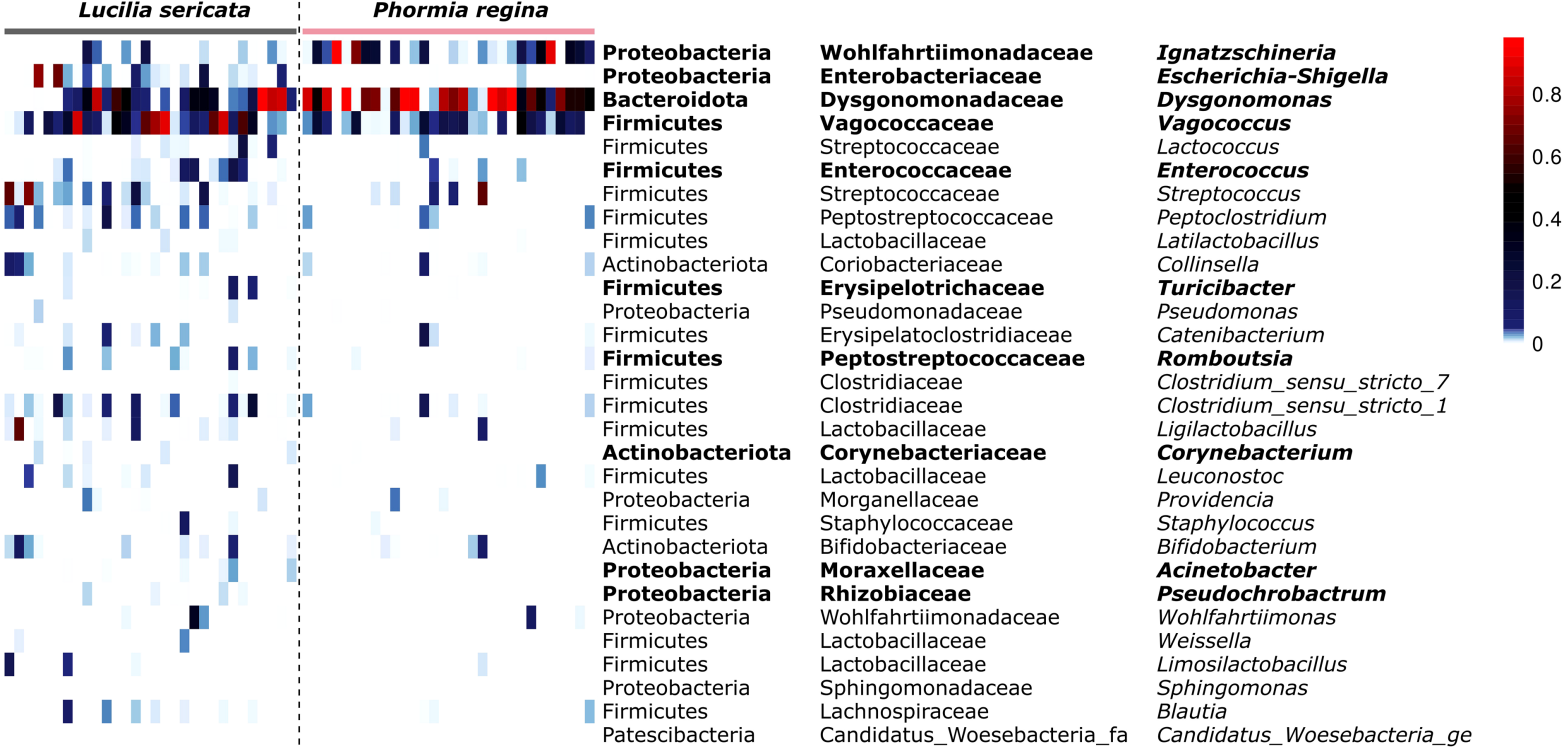
Heat-map of distribution and relative abundances for the 30 bacterial taxa identified as most informative (**Figure 2**), across all samples ordered by host species. Columns are individual samples grouped first by fly species. Rows list each taxon as Phylum; Family; Genus. Cell colours represent the proportion of reads contributed by each taxon in each sample.

To test for species-specific enrichment, we fitted a Tweedie generalized linear model for each of the top 30 taxa against host species and applied a False Discovery Rate correction. Ten taxa were significantly different in relative abundance between *L. sericata* and *P. regina* (FDR-adjusted *p* < 0.05; **Figure 3**; **Supplementary Table S4**). Notably, *Dysgonomonas* and *Ignatzschineria* were both enriched in *P. regina*, with *Dysgonomonas* comprising a median 65.3% of its microbiome (IQR: 13.4 – 86.2%) versus 7.4% in *L. sericata* (IQR: 0.2 – 38.1%; *p* = 0.009), and *Ignatzschineria* at 3.5% (IQR: 0.5 – 25.6%) versus essentially zero in *L. sericata* (IQR: 0.0 – 0.5%; *p* = 0.004). Conversely, *Vagococcus* was more abundant in *L. sericata* (median 12.1%, IQR: 2.5 – 38.2%) than in *P. regina* (5.9%, IQR: 1.1 – 17.5%; *p* = 0.014), and *Escherichia–Shigella* also showed higher levels in *L. sericata* (0.5%, IQR: 0.1 – 2.3%) relative to *P. regina* (0.0%, IQR: 0.0 – 0.1%; *p* = 0.005). Smaller but significant enrichments in *L. sericata* were observed for *Enterococcus* (0.3%, IQR: 0.1 – 1.1% vs. 0.0%; *p* = 0.025) and several low-abundance (<1% mean) genera including *Turicibacter*, *Romboutsia*, *Corynebacterium*, *Acinetobacter* and *Pseudochrobactrum*.

The remaining 20 taxa did not differ significantly between the two species, indicating that, beyond a core set of discriminatory bacteria, most genus-level groups were shared at similar abundances in *L. sericata* and *P. regina*.

## 4 Discussion

Our study demonstrates that blow fly bacterial microbiomes are primarily structured by host species identity rather than external environmental factors. Despite being collected from the same sampling sites, and exposed to similar environmental microbial reservoirs, *L. sericata* and *P. regina* maintain broadly distinct microbial communities (**Figures 1**, **3**). Beta diversity analyses (Morisita-Horn and Jaccard distance; **Figure 1**) confirm that host species identity was the strongest predictor of microbial composition (*p* = 0.001;**Supplementary Table S3**), with no significant effect observed for the other factors (*p* > 0.117; **Supplementary Table S3**). Several taxa that most strongly distinguished the two species, including *Ignatzschineria* and *Vagococcus*, are known from clinical settings, suggesting that predictable host-associated patterns may influence which pathogens blow flies regularly retain and transmit.

Random Forest analysis further supported this pattern (**Figure 2**); using taxon contribution scores for bacterial genus-level taxa allowed us to identify those genera whose abundance patterns most reliably distinguish sample categories. These scores reflect how strongly each taxon contributes to accurate classification of samples based on metadata variables like species, type, or location. While metadata variables such as Type and Location also yielded high raw classification accuracies, they relied on fewer taxa, mostly with low taxon contribution scores (**Figure 2A**), and none of which showed significant abundance differences (**Supplementary Table S4**). In contrast, the Species classifier was supported by numerous genus-level taxa (**Supplementary Table S4**) with consistently high taxon contribution scores, many of which also ranked highest in cumulative taxon contribution across all models (**Figure 2B**), indicating that blow fly species identity was broadly reflected in the microbiome. Because several of these taxa include known opportunistic pathogens, our results highlight that host identity strongly influences the bacterial groups that blow flies are likely to harbor and potentially transmit.

Although environmental exposure undoubtedly plays a role in determining microbiome composition in blow flies (12), our data show that host species identity is the primary driver under natural field conditions. Recent evidence from Burcham et al. (29) suggests that excluding insect access to cadavers reduces the abundance or completely eliminates specific bacterial taxa, especially *Ignatzschineria* and *Vagococcus*, from cadavers, reinforcing the ecological importance of blow fly-mediated transfer of specific bacterial taxa. However, the host specific signal in the blow fly microbiome is unlikely to be reproducible in lab-reared flies, where microbial acquisition is constrained by diet and rearing conditions. For example, Wohlfahrt et al. (11) found that when *L. sericata* and *P. regina* were reared on controlled diets in the lab, their microbiomes were not only compositionally distinct from those in our dataset (**Figure 3**), but also statistically indistinguishable from one another. Similarly, Iancu et al. (13) reported that lab-reared *L. sericata* tenerals harbored microbiomes dominated by *Lactobacillus*, *Acinetobacter*, and *Proteus*, a stark contrast to both Wohlfahrt et al.’s (11) lab population (characterized by *Vagococcus* and *Leuconostoc*) and our wild-caught individuals (*Vagococcus* and *Escherichia*-*Shigella*; **Figure 3**). These discrepancies suggest that, when environmental exposure is standardized, host species alone may not dictate microbiome composition. In contrast, field-collected flies consistently show species-associated patterns, which are most detectable when microbial acquisition occurs under natural ecological conditions that support pathogen exposure transmission.

For instance, wild-caught *P. regina* from Michigan consistently harbored *Ignatzschineria* and *Dysgonomonas* in the current study (**Figures 2C** and **3**)—a pattern also observed from field captured specimens in North Carolina by Deguenon et al. (15). Together, these findings suggest that host filtering of microbiomes is context-dependent and most detectable when environmental acquisition is not artificially constrained. Our Random Forest weighting approach therefore provided a targeted set of candidate genera, many of which are clinically relevant, offering a rigorous framework for future studies assessing their roles in pathogen persistence and transmission.

While *Vagococcus*, *Escherichia-Shigella*, *Ignatzschineria*, and *Dysgonomonas* were present in a majority of the samples across both species (**Figure 3**), Random Forest analysis showed that they reliably distinguish *L. sericata* from *P. regina*. This suggests that the two species do not simply acquire microbiota from a common environmental pool or that there are different feeding preferences that may affect their microbiomes. Our results suggest that they interact with and retain specific bacterial groups in a systematic, species-dependent manner, potentially through feeding differences (30). The structured differentiation of their microbiomes reflects not only microbial exposure during larval development but also ongoing modifications shaped by adult foraging behaviors and ecological interactions.

Microbial inheritance from the larval stage provides a foundation for adult microbiome composition but is likely modified by subsequent environmental exposures. It is well established that adult blow flies retain a substantial portion of their larval microbiome post-metamorphosis (11, 13, 16, 31, 32). However, larval microbiomes are not inherited in isolation; they reflect a combination of microbes from the oviposition substrate (2) as well as microbial members introduced by their mother during egg laying supporting both horizontal and vertical transmission (14). While these transgenerationally acquired microbes provide an initial template, their inheritance does not fully determine adult microbiome composition. Instead, the distinct foraging ecologies and feeding behaviors of *L. sericata* and *P. regina* post-eclosion likely reinforce species-specific microbiome differentiation, maintaining structured microbial assemblages despite environmental variability.

Adult foraging behaviors provide the strongest mechanism for microbiome differentiation, as they directly modify the microbiota after eclosion. *L. sericata* exhibits a broad foraging repertoire, in response to carrion volatiles, such as dimethyl disulfide and butan-1-ol (33) but also selectively visiting floral resources and bacterial volatile-based baits (34). In contrast, *P. regina* not only scavenges carrion but also frequently aggregates at animal feces (35). Given that blow flies engage in regurgitation-based feeding, these foraging differences likely reinforce microbiome divergence by continuously introducing new microbial exposures post-eclosion. The consistent presence of *Ignatzschineria* and *Dysgonomonas* in *P. regina* (**Figure 3**) may be partially attributed to its interactions with fecal substrates (35), which are known reservoirs of opportunistic pathogens. *Ignatzschineria*, in particular, has been frequently recovered from cases of human myiasis and necrotic wound infections (36, 37). Its ability to persist through metamorphosis (13, 14, 16) suggests pre-adaptations for colonizing blow fly guts — a feature that may facilitate its transmission in clinical settings.

Both *L. sericata* and *P. regina* can cause myiasis that leads to secondary infections (38). The capability to deliver pathogens to wounds raises important public health considerations. Notably, *Ignatzschineria* has gained increasing clinical attention due to recent reports describing the emergence of carbapenem-resistant strains (39), raising serious concerns about its potential role as an opportunistic pathogen with evolving resistance mechanisms. *Vagococcus*, that we found to be specifically associated with *L. sericata*, though often considered a commensal or opportunistic pathogen, has been increasingly reported in soft tissue infections (40)​. Clinical isolates have shown variable resistance to fluoroquinolones, tetracyclines, and macrolides, complicating treatment (41). By engaging in regurgitation-based feeding (1), blow flies may facilitate the persistence and spread of antibiotic-resistant strains, warranting deeper investigation.

A striking feature of our results is that *P. regina* harbors a microbiome characterized by fewer dominant bacterial taxa, particularly *Dysgonomonas* and *Ignatzschineria*, whereas *L. sericata* exhibits a more even distribution of microbial associates (**Figures 1**, **3**). This pattern suggests that the microbial community structure in *P. regina* is more specialized or constrained. One potential explanation can be attributed to the dietary specialization of *P. regina*, which rely heavily on carrion and feces (35). It selects for a subset of bacteria capable of thriving in these environments, leading to reduced overall diversity. Another possibility is that immune filtering contributes to this pattern, for example through stronger antimicrobial peptide activity or selective tolerance of specific microbial taxa. While our study did not directly measure immune responses, our findings generate testable hypotheses about how immunity may interact with ecology to structure blow fly microbiomes.

Evidence from the fruit fly genus *Drosophila* demonstrates that antimicrobial peptides and lysozymes directly regulate gut microbial composition (42), and broader reviews emphasize the role of innate immune pathways and gut barrier function in structuring insect microbiomes (43). In several insect groups, including termites (44, 45), cockroaches (46, 47), and beetles (48, 49), intestinal physicochemistry (e.g. pH, presence of food particles, and redox conditions) has been shown to influence microbial composition. Evidence in blow flies and house flies is comparatively sparse, but the compartmentalized gut of blow flies could impose comparable pH-mediated constraints. Within blow flies, host physiology and immunity have been recognized as key but understudied components of their microbe–host ecology (1). While our study did not directly measure immune responses, our results generate testable hypotheses about how immunity may interact with ecology to structure blow fly microbiomes.

A purely ecological explanation for the species-specificity of microbiomes can be competitive exclusion, where the colonization of the fly gut by say *Dysgonomonas* and *Ignatzschineria* suppresses the establishment of other taxa like *Vagococcus* and *Escherichia*-*Shigella*, or vice versa. More detailed examination assessing immune gene expression and bacterial interactions within *P. regina*’s gut could help determine whether host physiology actively constrains microbial diversity.

The timing of oviposition acts as a secondary driver of microbiome differentiation by shaping the microbial landscape encountered during larval development (2). *L. sericata*, which oviposits on carrion shortly after animal death (30), is exposed to an early-stage necrobiome dominated by facultative anaerobes that thrive under declining oxygen conditions (50). The detection of *Escherichia-Shigella*, which have been detected in lower gastrointestinal tract samples during both pre-bloat and end-bloat stages (51), aligns well with *L. sericata*’s early colonization of carrion. Similarly, *Vagococcus*, a lactic acid bacterium frequently found in decomposing animal tissues (52), may thrive under the lower pH conditions of early carrion decay.

In contrast, *P. regina* typically oviposits on remains at a later decomposition stage (30), characterized by lower redox potential (50) and increased bacterial proteolysis (53). This shift in microbial composition likely influences the taxa available for colonization, with *Dysgonomonas*, an amino acid-metabolizing genus frequently isolated from decaying animal tissues (54, 55), becoming more prominent. The enrichment of *Ignatzschineria* in *P. regina* across multiple datasets in various studies (15, 51) further supports the idea that its microbiome differentiation is shaped by its later interaction with a decompositional stage favoring anaerobic bacteria (29).

Collectively, our findings suggest that microbiome differentiation in blow flies arises from an interplay of (i) active microbiome modification through adult foraging behaviors, (ii) ecological exposure differences during larval colonization, and (iii) microbial inheritance from the larval stage. The relative contribution of each of these factors likely varies across individuals and populations, but their combined effect ensures that species-specific microbial structuring is not merely a byproduct of shared environmental exposure.

The divergence between wild and lab-reared microbiomes highlights the importance of natural foraging ecologies in shaping bacterial communities. Given that blow flies frequently interact with decomposing tissues, wounds, and fecal matter, their microbiomes may influence not only decomposition dynamics but also the transmission of opportunistic or antibiotic-resistant pathogens.

By demonstrating that host species identity consistently overrides environmental variation, our study establishes a firm baseline for understanding blow fly–microbiome associations. This finding naturally raises questions about mechanism, generality, and consequence. Mechanistically, a limitation of the present design is that it cannot directly resolve whether immune filtering, gut physiology, or microbial inheritance underlie these species-specific differences; rather, it sets the stage for targeted immunological and physiological assays to test these mechanisms. Future studies are needed that use larger fly data sets over larger spatial scales and more comprehensive environmental variables to increase statistical power to detect the relative roles of these other factors (e.g., immunological, physiological, environmental, ecological) in structuring blow fly microbiomes. Such studies would provide future advances in understanding the interfaces of blow fly biology, ecology, landscape and microbiomes to insect immunology and physiology. In terms of generality, our geographic and seasonal scope represents another limitation that creates an opportunity for replication across regions and timeframes to evaluate the stability of the species effect. Finally, the ecological and clinical consequences of these species-specific microbiomes—particularly the enrichment of taxa with opportunistic pathogenic potential—cannot be resolved with 16S profiles alone, but this highlights the need for gnotobiotic and culture-based viability studies, strain-level metagenomics, and host transcriptomics, as well as experiments where microbiome composition can be experimentally controlled. In this sense, the limitations of the current study are not weaknesses of inference but instead define the most compelling next steps for the field: moving from species-level pattern to mechanistic understanding and predictive insight.

## Data Availability

The datasets presented in this study can be found in online repositories. The names of the repository/repositories and accession number(s) can be found below: https://www.ncbi.nlm.nih.gov/genbank/, PRJNA1292902.
