# Supplementary material for: Host-specific microbiomes of blow flies: ecological drivers and implications for pathogen carriage
Source: Front Immunol. 2025 Nov 27;16:1673934. doi: 10.3389/fimmu.2025.1673934 (PMC12695767; doi:10.3389/fimmu.2025.1673934)
Supplement: Supplementary file 1 [file Table1.docx]

Supplementary Material

# Supplementary Figures and Tables

## Supplementary Figures


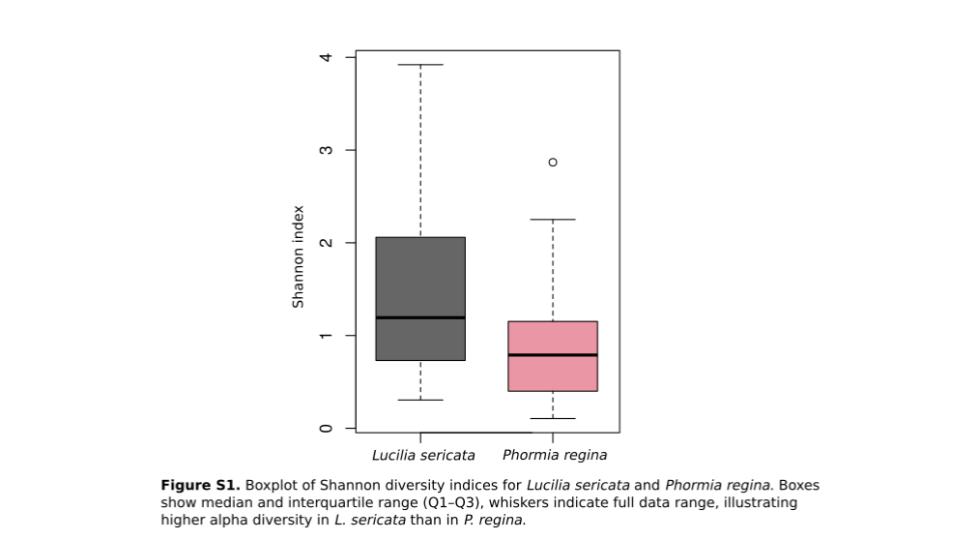


**Supplementary Figure 1.** Boxplot of Shannon diversity indices for *Lucilia sericata* and *Phormia regina*. Boxes show median and interquartile range (Q1—Q30, whiskers indicate full data range, illustrating higher alpha diversity in *L. sericata* than in *P. regina*

*
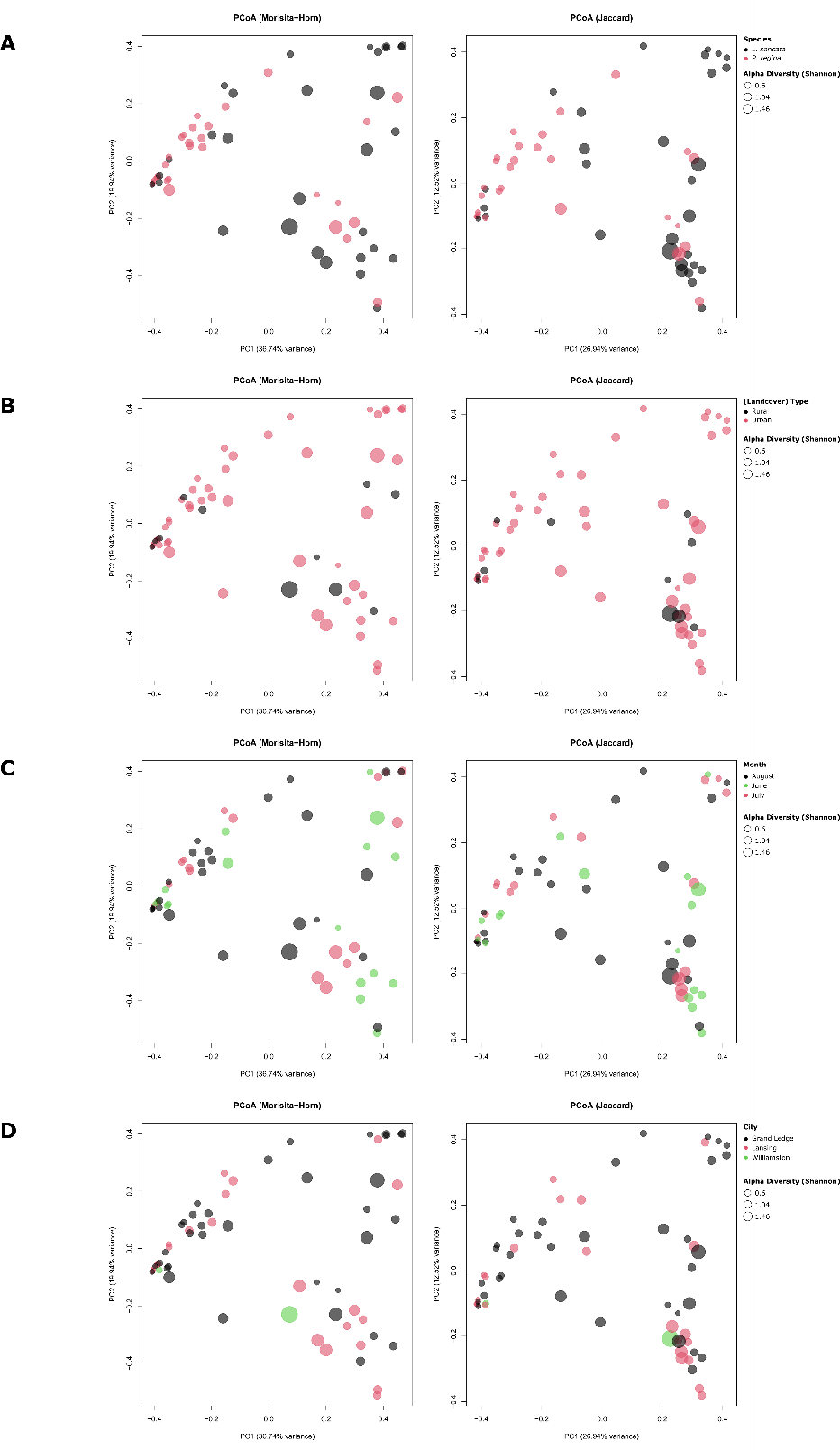
*

**Supplementary Figure 2.** Principal Coordinate Analysis of blowfly microbiome composition using Morisita–Horn and Jaccard distance metrics. Each point represents an individual sample, colored by species (A), (landcover) Type (B), Month (year: 2017, C) and City (D) and scaled by Shannon diversity (larger points indicate higher diversity). Axes show the first two principal coordinates and the percentage of total variation they explain.

# Supplementary Tables

**Table S1.** Metadata for blow-fly microbiome sequencing samples, including collection site (city and distance), Date, Month, Species (fly species: *L, sericata*, and *P. regina*) and sex, “Processor”, “Type” (Landcover type), and the number of 16S rRNA gene contigs at the end of quality screening.

|  | City | Location | Date | Month | Species | Sex | Processor | Type | QC-Trimmed reads |
| --- | --- | --- | --- | --- | --- | --- | --- | --- | --- |
| KB00037 | Williamston | North1km | 08/14/17 | Aug | *L. sericata* | F | Babcock | Urban | 33175 |
| KB00055 | Williamston | South10km | 08/14/17 | Aug | *L. sericata* | F | Babcock | Rural | 55338 |
| KB00447 | Lansing | West1km | 07/01/17 | Jul | *L. sericata* | M | Babcock | Urban | 51007 |
| KB00454 | Lansing | West1km | 07/01/17 | Jul | *L. sericata* | F | Babcock | Urban | 5532 |
| KB00478 | Lansing | East1km | 07/01/17 | Jul | *L. sericata* | M | Babcock | Urban | 45112 |
| KB00485 | Lansing | East1km | 07/01/17 | Jul | *L. sericata* | F | Babcock | Urban | 9536 |
| KB00545 | Lansing | North1km | 07/01/17 | Jul | *L. sericata* | M | Babcock | Urban | 38716 |
| KB00552 | Lansing | North1km | 07/01/17 | Jul | *L. sericata* | F | Babcock | Urban | 1149 |
| KB00651 | Lansing | North1km | 06/07/17 | Jun | *L. sericata* | M | Babcock | Urban | 57961 |
| KB00657 | Lansing | North1km | 06/07/17 | Jun | *L. sericata* | F | Babcock | Urban | 36233 |
| KB00677 | Lansing | East1km | 08/03/17 | Aug | *L. sericata* | M | Babcock | Urban | 34230 |
| KB00682 | Lansing | East1km | 08/03/17 | Aug | *L. sericata* | F | Babcock | Urban | 32251 |
| KB00827 | Lansing | West1km | 08/03/17 | Aug | *L. sericata* | M | Babcock | Urban | 22099 |
| KB00833 | Lansing | West1km | 08/03/17 | Aug | *L. sericata* | F | Babcock | Urban | 108 |
| KB01378 | GrandLedge | North1km | 06/10/17 | Jun | *L. sericata* | M | Woods | Urban | 6336 |
| KB01379 | GrandLedge | North1km | 06/10/17 | Jun | *L. sericata* | F | Woods | Urban | 3500 |
| KB01392 | GrandLedge | South1km | 06/15/17 | Jun | *L. sericata* | M | Smiles | Urban | 7413 |
| KB01393 | GrandLedge | South1km | 06/15/17 | Jun | *L. sericata* | F | Smiles | Urban | 4820 |
| KB01429 | GrandLedge | West1km | 08/08/17 | Aug | *L. sericata* | F | Patrick | Urban | 40239 |
| KB01435 | GrandLedge | West1km | 08/08/17 | Aug | *L. sericata* | M | Patrick | Urban | 33687 |
| KB01480 | GrandLedge | East1km | 06/15/17 | Jun | *L. sericata* | M | Himmel | Urban | 3340 |
| KB01486 | GrandLedge | East1km | 06/15/17 | Jun | *L. sericata* | F | Himmel | Urban | 114 |
| KB01532 | GrandLedge | West10km | 08/08/17 | Aug | *L. sericata* | F | Smiles | Rural | 1011 |
| KB01553 | GrandLedge | West1km | 07/05/17 | Jul | *L. sericata* | M | Weatherbee | Urban | 48399 |
| KB01556 | GrandLedge | West1km | 07/05/17 | Jul | *L. sericata* | F | Weatherbee | Urban | 3538 |
| KB01581 | GrandLedge | South1km | 08/08/17 | Aug | *L. sericata* | M | Himmel | Urban | 12114 |
| KB01587 | GrandLedge | South1km | 08/08/17 | Aug | *L. sericata* | F | Himmel | Urban | 4605 |
| KB01605 | GrandLedge | East1km | 08/08/17 | Aug | *L. sericata* | M | Scott | Urban | 6670 |
| KB01607 | GrandLedge | East1km | 08/08/17 | Aug | *L. sericata* | F | Scott | Urban | 16697 |
| KB01728 | GrandLedge | East10km | 06/10/17 | Jun | *L. sericata* | M | Himmel | Rural | 34589 |
| KB01729 | GrandLedge | East10km | 06/10/17 | Jun | *L. sericata* | F | Himmel | Rural | 31964 |
| KB01791 | GrandLedge | South10km | 08/08/17 | Aug | *L. sericata* | M | Smiles | Rural | 36094 |
| KB01794 | GrandLedge | South10km | 08/08/17 | Aug | *L. sericata* | F | Smiles | Rural | 52348 |
| KB00459 | Lansing | West1km | 07/01/17 | Jul | *P. regina* | M | Babcock | Urban | 41635 |
| KB00466 | Lansing | West1km | 07/01/17 | Jul | *P. regina* | F | Babcock | Urban | 34536 |
| KB00508 | Lansing | East1km | 07/01/17 | Jul | *P. regina* | M | Babcock | Urban | 76912 |
| KB00515 | Lansing | East1km | 07/01/17 | Jul | *P. regina* | F | Babcock | Urban | 62962 |
| KB00532 | Lansing | North1km | 07/01/17 | Jul | *P. regina* | M | Babcock | Urban | 46963 |
| KB00539 | Lansing | North1km | 07/01/17 | Jul | *P. regina* | F | Babcock | Urban | 67548 |
| KB00665 | Lansing | North1km | 06/07/17 | Jun | *P. regina* | M | Babcock | Urban | 44833 |
| KB00671 | Lansing | North1km | 06/07/17 | Jun | *P. regina* | F | Babcock | Urban | 27681 |
| KB00698 | Lansing | East1km | 08/03/17 | Aug | *P. regina* | M | Babcock | Urban | 53628 |
| KB00704 | Lansing | East1km | 08/03/17 | Aug | *P. regina* | F | Babcock | Urban | 54736 |
| KB00815 | Lansing | West1km | 08/03/17 | Aug | *P. regina* | M | Babcock | Urban | 51219 |
| KB00821 | Lansing | West1km | 08/03/17 | Aug | *P. regina* | F | Babcock | Urban | 43976 |
| KB01380 | GrandLedge | North1km | 06/10/17 | Jun | *P. regina* | M | Woods | Urban | 41123 |
| KB01386 | GrandLedge | North1km | 06/10/17 | Jun | *P. regina* | F | Woods | Urban | 33494 |
| KB01396 | GrandLedge | South1km | 06/15/17 | Jun | *P. regina* | M | Smiles | Urban | 35783 |
| KB01402 | GrandLedge | South1km | 06/15/17 | Jun | *P. regina* | F | Smiles | Urban | 454 |
| KB01421 | GrandLedge | West1km | 08/08/17 | Aug | *P. regina* | F | Patrick | Urban | 1195 |
| KB01423 | GrandLedge | West1km | 08/08/17 | Aug | *P. regina* | M | Patrick | Urban | 42031 |
| KB01492 | GrandLedge | East1km | 06/15/17 | Jun | *P. regina* | M | Himmel | Urban | 8629 |
| KB01498 | GrandLedge | East1km | 06/15/17 | Jun | *P. regina* | F | Himmel | Urban | 807 |
| KB01518 | GrandLedge | West10km | 08/08/17 | Aug | *P. regina* | M | Smiles | Rural | 53799 |
| KB01524 | GrandLedge | West10km | 08/08/17 | Aug | *P. regina* | F | Smiles | Rural | 56897 |
| KB01541 | GrandLedge | West1km | 07/05/17 | Jul | *P. regina* | M | Weatherbee | Urban | 37830 |
| KB01547 | GrandLedge | West1km | 07/05/17 | Jul | *P. regina* | F | Weatherbee | Urban | 37199 |
| KB01569 | GrandLedge | South1km | 08/08/17 | Aug | *P. regina* | M | Himmel | Urban | 54311 |
| KB01575 | GrandLedge | South1km | 08/08/17 | Aug | *P. regina* | F | Himmel | Urban | 35670 |
| KB01593 | GrandLedge | East1km | 08/08/17 | Aug | *P. regina* | M | Scott | Urban | 57918 |
| KB01599 | GrandLedge | East1km | 08/08/17 | Aug | *P. regina* | F | Scott | Urban | 47859 |
| KB01698 | GrandLedge | West10km | 07/05/17 | Jul | *P. regina* | M | Patrick | Rural | 37076 |
| KB01704 | GrandLedge | West10km | 07/05/17 | Jul | *P. regina* | F | Patrick | Rural | 60038 |
| KB01733 | GrandLedge | East10km | 06/10/17 | Jun | *P. regina* | M | Himmel | Rural | 28590 |
| KB01739 | GrandLedge | East10km | 06/10/17 | Jun | *P. regina* | F | Himmel | Rural | 16688 |

**Table S2.** Results of Wilcoxon rank-sum and Kruskal–Wallis tests comparing Shannon diversity across metadata factors; only species identity showed a significant effect (p = 0.0039), while city, location, month, gender, and type did not (all p > 0.05).

| Factor | *p* value | Test |
| --- | --- | --- |
| Species | 0.003906082 | Wilcoxon rank-sum |
| City | 0.702525187 | Kruskal-Wallis |
| Location | 0.945755772 | Kruskal-Wallis |
| Month | 0.851687207 | Kruskal-Wallis |
| Gender | 0.263030823 | Wilcoxon rank-sum |
| Processor | 0.335982062 | Kruskal-Wallis |
| Type | 0.450090982 | Wilcoxon rank-sum |

**Table S3.** PERMANOVA results for each metadata variable on blowfly microbiome composition, using Morisita–Horn and Jaccard distance metrics. For each factor, the table lists degrees of freedom (Df), sum of squares (SumOfSqs), proportion of variance explained (R²), residual R², F-statistic (F), and permutation-based P-value (P).

| **Variable** | **Metric** | **Df** | **SumOfSqs** | **R2** | **Residual R2** | **F** | **P** |
| --- | --- | --- | --- | --- | --- | --- | --- |
| City | Morisita-Horn | 2 | 0.744 | 0.045 | 0.955 | 1.342 | 0.189 |
|  | Jaccard | 2 | 0.757 | 0.037 | 0.963 | 1.090 | 0.332 |
| Location | Morisita-Horn | 6 | 2.214 | 0.134 | 0.866 | 1.366 | 0.117 |
|  | Jaccard | 6 | 2.240 | 0.109 | 0.891 | 1.081 | 0.314 |
| Month | Morisita-Horn | 2 | 0.617 | 0.037 | 0.963 | 1.106 | 0.335 |
|  | Jaccard | 2 | 0.632 | 0.031 | 0.969 | 0.905 | 0.559 |
| Species | Morisita-Horn | 1 | 1.795 | 0.109 | 0.891 | 7.064 | 0.001 |
|  | Jaccard | 1 | 1.812 | 0.088 | 0.912 | 5.613 | 0.001 |
| Sex | Morisita-Horn | 1 | 0.320 | 0.019 | 0.981 | 1.146 | 0.297 |
|  | Jaccard | 1 | 0.436 | 0.021 | 0.979 | 1.257 | 0.233 |
| Processor | Morisita-Horn | 8 | 1.835 | 0.111 | 0.889 | 0.796 | 0.818 |
|  | Jaccard | 8 | 2.383 | 0.116 | 0.884 | 0.837 | 0.849 |
| Type | Morisita-Horn | 1 | 0.352 | 0.021 | 0.979 | 1.263 | 0.245 |
|  | Jaccard | 1 | 0.401 | 0.020 | 0.980 | 1.156 | 0.277 |

**Table S4.** Top 30 taxa selected by cumulative Random-Forest importance (MDG × OOB). Columns list adjusted *P*-values from Tweedie GLMs (var.power = 1.5); values in red (< 0.05) indicate taxa whose relative abundance differs significantly between *Lucilia sericata* and *Phormia regina* (Species column) or between sample landcover types (Urban vs Rural)

| **Taxon** | **Species** | **Type** |
| --- | --- | --- |
| Proteobacteria;Gammaproteobacteria;Cardiobacteriales;Wohlfahrtiimonadaceae;Ignatzschineria; | 0.004 | 0.417 |
| Proteobacteria;Gammaproteobacteria;Enterobacterales;Enterobacteriaceae;Escherichia-Shigella; | 0.005 | 0.660 |
| Bacteroidota;Bacteroidia;Bacteroidales;Dysgonomonadaceae;Dysgonomonas; | 0.009 | 0.993 |
| Firmicutes;Bacilli;Lactobacillales;Vagococcaceae;Vagococcus; | 0.014 | 0.346 |
| Firmicutes;Bacilli;Lactobacillales;Streptococcaceae;Lactococcus; | 0.122 | 0.941 |
| Firmicutes;Bacilli;Lactobacillales;Enterococcaceae;Enterococcus; | 0.025 | 0.346 |
| Firmicutes;Bacilli;Lactobacillales;Streptococcaceae;Streptococcus; | 0.397 | 0.375 |
| Firmicutes;Clostridia;Peptostreptococcales-Tissierellales;Peptostreptococcaceae;Peptoclostridium; | 0.066 | 0.346 |
| Firmicutes;Bacilli;Lactobacillales;Lactobacillaceae;Latilactobacillus; | 0.096 | 0.346 |
| Actinobacteriota;Coriobacteriia;Coriobacteriales;Coriobacteriaceae;Collinsella; | 0.563 | 0.346 |
| Firmicutes;Bacilli;Erysipelotrichales;Erysipelotrichaceae;Turicibacter; | 0.016 | 0.587 |
| Proteobacteria;Gammaproteobacteria;Pseudomonadales;Pseudomonadaceae;Pseudomonas; | 0.098 | 0.587 |
| Firmicutes;Bacilli;Erysipelotrichales;Erysipelatoclostridiaceae;Catenibacterium; | 0.714 | 0.486 |
| Firmicutes;Clostridia;Peptostreptococcales-Tissierellales;Peptostreptococcaceae;Romboutsia; | 0.016 | 0.647 |
| Firmicutes;Clostridia;Clostridiales;Clostridiaceae;Clostridium_sensu_stricto_7; | 0.438 | 0.486 |
| Firmicutes;Clostridia;Clostridiales;Clostridiaceae;Clostridium_sensu_stricto_1; | 0.122 | 0.929 |
| Firmicutes;Bacilli;Lactobacillales;Lactobacillaceae;Ligilactobacillus; | 0.171 | 0.587 |
| Actinobacteriota;Actinobacteria;Corynebacteriales;Corynebacteriaceae;Corynebacterium; | 0.014 | 0.796 |
| Firmicutes;Bacilli;Lactobacillales;Lactobacillaceae;Leuconostoc; | 0.164 | 0.796 |
| Proteobacteria;Gammaproteobacteria;Enterobacterales;Morganellaceae;Providencia; | 0.681 | 0.796 |
| Firmicutes;Bacilli;Staphylococcales;Staphylococcaceae;Staphylococcus; | 0.119 | 0.587 |
| Actinobacteriota;Actinobacteria;Bifidobacteriales;Bifidobacteriaceae;Bifidobacterium; | 0.547 | 0.587 |
| Proteobacteria;Gammaproteobacteria;Pseudomonadales;Moraxellaceae;Acinetobacter; | 0.009 | 0.796 |
| Proteobacteria;Alphaproteobacteria;Rhizobiales;Rhizobiaceae;Pseudochrobactrum; | 0.002 | 0.375 |
| Proteobacteria;Gammaproteobacteria;Cardiobacteriales;Wohlfahrtiimonadaceae;Wohlfahrtiimonas; | 0.547 | 0.587 |
| Firmicutes;Bacilli;Lactobacillales;Lactobacillaceae;Weissella; | 0.066 | 0.674 |
| Firmicutes;Bacilli;Lactobacillales;Lactobacillaceae;Limosilactobacillus; | 0.098 | 0.587 |
| Proteobacteria;Alphaproteobacteria;Sphingomonadales;Sphingomonadaceae;Sphingomonas; | 0.183 | 0.886 |
| Firmicutes;Clostridia;Lachnospirales;Lachnospiraceae;Blautia; | 0.119 | 0.417 |
| Patescibacteria;Microgenomatia;Candidatus_Woesebacteria;Candidatus_Woesebacteria_fa;Candidatus_Woesebacteria_ge; | 0.961 | 0.346 |
